# CUMULATIVE DIS/ADVANTAGE AND HEALTH TRAJECTORY IN LATE LIFE: A COMPARISON BETWEEN FOUR COUNTRIES

**DOI:** 10.1093/geroni/igz038.3043

**Published:** 2019-11-08

**Authors:** Peiyi Lu, Mack Shelley

**Affiliations:** Iowa State University, Ames, Iowa, United States

## Abstract

Cumulative Dis/Advantage (CDA) theory concerns how societal structure influences individual developmental and health trajectory across the life span, but few studies have applied CDA in the international setting with gender comparisons. This study provides a cross-national perspective to test CDA in explaining health inequality between developing and developed countries. Cross-sectional harmonized data from the international Health Retirement Study-series (United States, China, Mexico, and England) in 2013-2014 were used (n=97,978). Four health indicators were included: self-reported health, depressive symptoms, functional ability, and memory. Sociodemographic information and health behaviors were used as covariates. Regression models were fitted to examine the moderation roles of country and gender in health trajectory in later life. Results indicate self-reported health and mental health remained steady while functional ability and memory declined across late life span. Older Chinese and Mexican respondents had poorer health status than their British and American counterparts consistently except for memory in the Mexican data. Cumulative health gaps between developing and developed countries existed only for functional ability. Females in the four countries had poorer health than their male counterparts except their memory status. We conclude that CDA explains only the temporal decline of functional ability and its increasing gaps in later life between countries. Health inequality between countries could be attributed to the limited availability of healthcare and social resources in developing countries. However, other health status, including general health and mental health, depend more on individuals’ intrinsic capacity and human agency.

